# Determining the Factors Associated with Cardiovascular Disease Recurrence: Tehran Lipid and Glucose Study

**Published:** 2017-07

**Authors:** Samira Taravatmanesh, Davood Khalili, Soheila Khodakarim, Samaneh Asgari, Farzad Hadaegh, Fereidon Azizi, Siamak Sabour

**Affiliations:** 1 *Department of Epidemiology, School of Health, Shahid Beheshti University of Medical Sciences, Tehran, Iran.*; 2 *Prevention of Metabolic Disorders Research Center, Research Institute for Endocrine Sciences, Shahid Beheshti University of Medical Sciences, Tehran, Iran.*; 3 *Endocrine Research Center, Research Institute for Endocrine Sciences, Shahid Beheshti University of Medical Sciences, Tehran, Iran.*; 4 *Safety Promotions and Injuries Prevention Research Centre, Shahid Beheshti University of Medical Sciences, Tehran, Iran.*

**Keywords:** *Cardiovascular diseases*, *Recurrence*, *Risk factors*

## Abstract

**Background: **Several studies have emphasized the importance of cardiovascular disease (CVD) prevention. However, there is a dearth of data on the prevention of cardiovascular disease recurrence. The present study was the 1st in Iran to evaluate factors associated with CVD recurrence.

**Methods:** This prospective cohort study was conducted on 483 subjects (> 30 years old) with a history of CVD who participated in the Tehran Lipid and Glucose Study and were followed up for 12 years (1999–2012). The relationships between the most important established risk factors for CVD and CVD recurrence were evaluated.

**Results:** Totally, 258 (53.4%) men and 225(46.5%) women at a mean age of 59.2 ± 10.7 years were recruited in the study. Our results showed that over the 12-year follow-up, the incidence of a recurrent event (per 100 person-years) was 48.5. Further, after controlling the possible confounding factors, the following variables had a significant relationship with CVD recurrence: age (HR = 1.02; p value = 0. 001), male sex (HR = 1.4; p value = 0.012), smoking (HR = 1.7; p value = 0.004), and increased fasting blood sugar (HR = 2.1; p value = 0.001).

**Conclusion:** We found that the established variables in the development of CVD (i.e., age, sex, and smoking) played an important role in the risk of CVD recurrence.

## Introduction

Cardiovascular disease (CVD) is one of the most common diseases in the world.^1^ While many studies have underscored the importance of CVD prevention, few studies have highlighted the significance of the prevention of its recurrence.^2^ Indeed, for all the studies having evaluated the risk factors associated with CVD, precious little has been done to investigate whether or not the established risk factors for CVD may predict the recurrence of this disease.^3^ Hypertension, hyperlipidemia, diabetes, smoking, age, and sex are generally accepted as risk factors for CVD.^4^^, ^^5^ Research has demonstrated that the established CVD risk factors play an important role in the recurrence of CVD.^6^

As yet, no study has been done on CVD recurrence in Iran. Accordingly, we sought to determine the factors associated with the recurrence of CVD using the population under study in the Tehran Lipid and Glucose Study (TLGS). The CVD in this population is defined as a combination of myocardial infarction, angina, and stroke. 

## Methods

To conduct the present research, we drew upon the data from the TLGS. This study has been investigating noncommunicable diseases such as CVD and their associated factors since 1999, using a sample of 15005 people (3 ≤ years old) in Tehran. Another 3550 participants were recruited in the 2nd phase of examination 3 years later, and the study is currently ongoing.^7^

***Exposure and Possible Confounding Factors***

These factors encompassed history of hypertension or taking antihypertensive drugs, systolic blood pressure > 140 mm Hg, diastolic blood pressure > 90 mmHg, history of diabetes or taking antidiabetic drugs, increased fasting blood sugar > 126 mg/dL, history of hyperlipidemia or taking lipid medication, increased levels of blood lipids (total cholesterol > 200 mg/dL, triglyceride > 150 mg/dL, or high-density lipoprotein < 40 mg/dL), daily smoking (smoking regularly or occasionally at the time of completing the questionnaire or before that and not smoking regularly or occasionally at the time of completing the questionnaire), taking cardiovascular drugs (taking any type of CVD drugs prescribed by doctors such as beta-blockers, angiotensin-converting inhibitors, and dipyridamole), and history of premature CVD in the family (history of heart attack or stroke or sudden death in the 1st-degree male family members < 55 years of age and the 1st-degree female family members < 65 years old).

The variables of low-density lipoprotein and consuming aspirin and warfarin were excluded due to the lack of the unavailability of the associated data, lack of proportional hazard assumption, and lack of recurrence data, respectively.

In the present study, CVD refers to coronary heart disease (angina and myocardial infarction) and cerebrovascular occurrences. Also, recurrent CVD refers to any recurrence of CVD (during the follow-up) in patients whose 1st occurrence of the disease has become stable and also 28 days have passed from the 1st event resulting in hospitalization (unstable angina, myocardial infarction, positive coronary angiography, death due to cardiac ischemia, cerebrovascular accidents, and death due to stroke). The patients were followed up on an annual basis and after gathering documentation from the hospital or from witnesses. The information is evaluated in the TLGS Research Center by a committee, comprising internists, cardiologists, epidemiologists, physicians in charge of data collection and, if necessary, related experts. Should any event befall the patients, the committee is tasked with evaluating it. 

The details of the outcome measurements were published in advance.^8^

**Figure 1:**
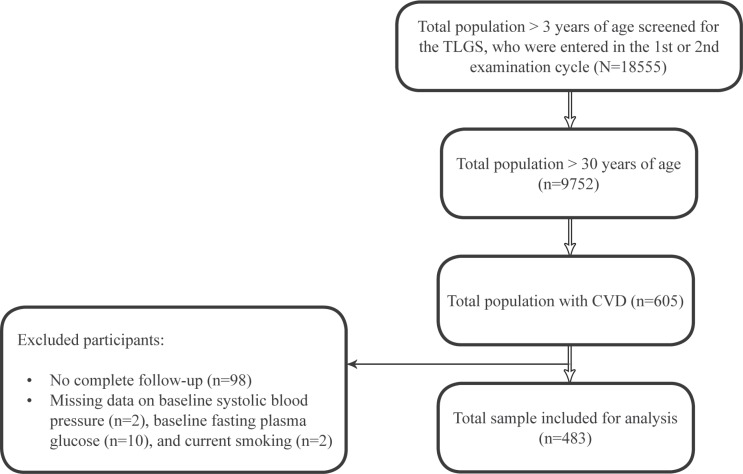
Flow chart of the study population

TLGS, Tehran Lipid and Glucose Study; CVD, Cardiovascular disease

Among all the 18555 people entered into the TLGS in phases 1 and 2, a total of 483 people (Figure 1) had a history of CVD and were followed up for 12 years (1999–2001 to 20 March 2012) and 227 patients experienced CVD recurrence. Considering α = 0.05, hazard ratio (HR) = 1.6, and 95% confidence interval (CI), the study power was also estimated to be 88%. For the association between baseline established risk factors and CVD recurrence, the univariate and multiple Cox proportional hazard models were used. Thus, proportionality (using a graphical method to check the proportional hazard model) and collinearity for the research variables were checked. As was explained above, aspirin was excluded owing to the absence of the proportional hazard assumption for this medicine. Variables with a p value ≤ 0.25 in the univariate analysis were entered into the multiple analyses.^9^^-^^10^ The STATA^11^ software was used for data analysis. 

## Results

Initially, 483 individuals over 30 years of age (225 women and 258 men) with a history of CVD were entered in the study. The mean age of the subjects was 59.3 years, with an SD of 10.5. Ninety-eight individuals with a history of CVD were excluded because of their incomplete follow-up period. To compare the features of this group with the 483 cases who completed the follow-up period, we used the independent *t*-test and the χ^2^ test. The results showed no significant differences between most of the variables of the 2 groups. Nonetheless, regarding age, there was a significant difference (p value < 0.001). Over the 12-year follow-up, the incidence of a recurrent event (per 100 person-years) was 48.5. The baseline characteristics of the included patients are depicted in Table 1.

The Cox regression model was applied to assess the relationship between each variable and CVD recurrence. The multiple Cox regression model was then utilized to examine the factors related to the recurrence of CVD in order to control the probable confounding variables. Then, the variables with a p value ≤ 0.25 in the univariate analysis were entered in the multiple analysis.Therefore, given a p value ≤ 0.25, the remaining variables in the final model comprised age, sex, smoking , history of hypertension, history of diabetes, history of CVD (father), systolic blood pressure, increased fasting blood sugar, angiotensin-converting inhibitors, antihypertensive drugs, and antidiabetic drugs. The results of the univariate Cox proportional HR and the multiple Cox proportional HR are shown in Table 2. The results revealed that after controlling for the possible confounding factors, there were significant relationships between CVD occurrence and age (HR = 1.02; p value < 0. 001 ), male sex (HR = 1.4; p value = 0.012), smoking (HR = 1.7; p value = 0.004 ), and increased fasting blood sugar (HR = 2.1; p value < 0.001).

**Figure 2:**
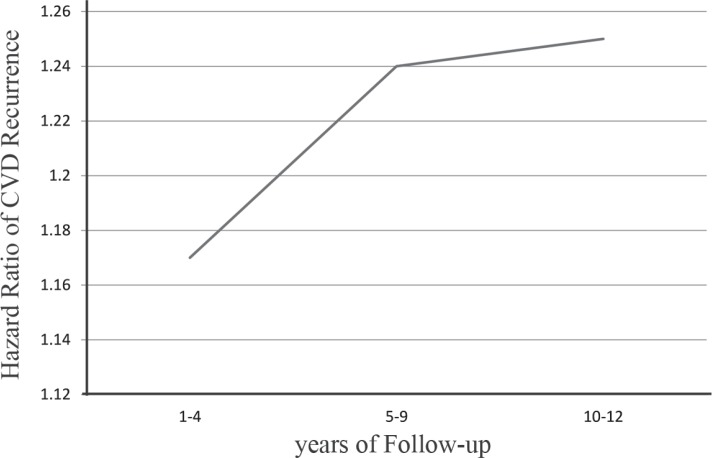
Hazard ratio of cardiovascular disease (CVD) recurrence in men versus women adjusted for age in 12 years of follow-up.

The HR for CVD recurrence in men versus women during the 12 years of follow-up is shown in (Figure 2). 

**Figure 3:**
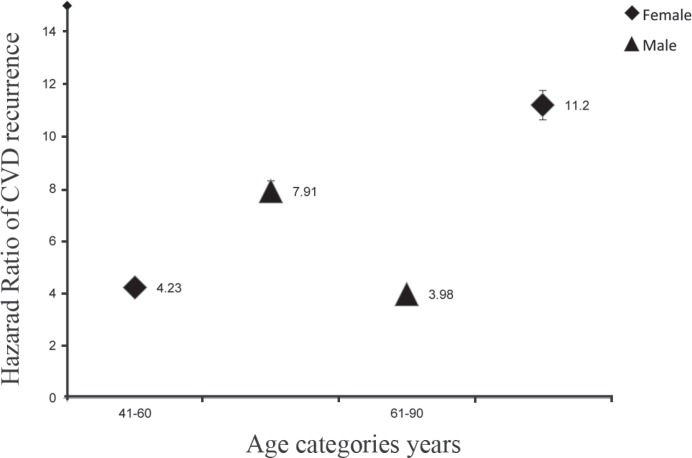
Hazard ratio of cardiovascular (CVD) recurrence by sex in different age categories after 12 years of follow-up (30–40 years as reference category).

The HR of increasing age for CVD recurrence according to sex is illustrated in Figure 3.

**Figure 4:**
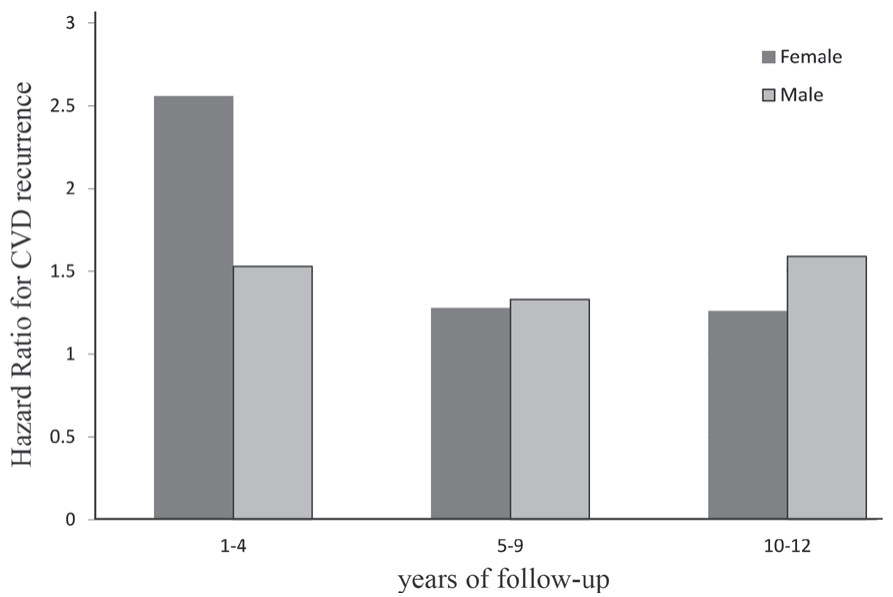
Hazard ratio of cardiovascular disease (CVD) recurrence in men versus women with a history of diabetes adjusted for age.

The HR of a history of diabetes for developing CVD recurrence according to sex is shown in Figure 4. Compared to the women, the diabetic men had a low risk of CVD recurrence in the 1st 5 years of the follow-up. This ratio was reversed at the end of the follow-up, with the risk of CVD recurrence rising in the diabetic men

**Table 1:** Baseline characteristics of the included patients (N=483)*

Variables	Value	Recurrence (density incidence rate per 100 person-years)	Variables	Value	Recurrence (density incidence rate per 100 person-years)
Age (y)	10.7±59.2		Triglycerides		
Gender			< 150 mg/dL	179 (37.1)	45.2
Male	258 (53.4)	42.9	≥ 150 mg/dL	304 (62.9)	42.9
Women	225 (46.5)	46.7	High-density lipoprotein		
Current smoking			≥ 40 mg/dL	223 (46.4)	21.9
Yes	75 (15.5)	43.0	< 40 mg/dL	258 (53.6)	68.5
No	408 (84.4)	44.7	Fasting blood sugar		
Hypertension			< 126 mg/dL	379 (78.5)	51.9
Yes	237 (49.1)	50.6	≥ 126 mg/dL	104 (21.5)	41.7
No	245 (50.8)	39.2	Drug History		
Diabetes Mellitus			Beta-Blocker		
Yes	131 (27.4)	50.6	Yes	222 (45.9)	49.1
No	346 (72.5)	41.6	No	261 (54.0)	40.7
Dyslipidemia			ACE Inhibitor		
Yes	234 (49.1)	48.5	Yes	67 (13.8)	77.7
No	242 (50.8)	40.8	No	416 (86.1)	41.1
History of Premature CVD			Dipyridamole		
Yes	67 (14.2)	64.4	Yes	29 (6.0)	48.2
No	402 (85.7)	42.4	No	454 (94.0)	46.9
History of Premature CVD			Anti-Lipid		
Yes	67 (14.6)	52.1	Yes	53 (10.9)	56.5
No	393 (85.4)	43.1	No	430 (89.1)	43.1
Systolic blood pressure (mmHg)			Antihypertensive		
< 140 mg/dL	320 (66.3)	45.5	Yes	201(41.6)	50.7
≥ 140 mg/dL	163 (33.7)	43.7	No	282 (58.3)	40.0
Diastolic blood pressure (mmHg)			Antidiabetic		
< 90 mg/dL	382 (79.1)	47.4	Yes	89 (18.4)	58.3
≥ 90 mg/dL	101 (20.9)	45.9	No	394 (81.5)	41.1
Total cholesterol					
< 200 mg/dL	133 (27.5)	43.7			
≥ 200 mg/dL	350 (72.5)	46.6			

CVD, Cardiovascular disease; ACE, Angiotensin-converting enzyme

* Data are presented as means±SDs or numbers (%).

**Table 2:** Univariate and multiple Cox models for CVD recurrence

Variable	Univariate Analysis		Multivariate Analysis	
	HR (95% CI)	P value	HR (95% CI)	P value
Age (y)	1.02 (1.01-1.03)	< 0.001	1.02 ( 1.01-1.03)	< 0.001
Gender				
Female	Reference			
Male	1.4	0.009	1.45 (1.08-1.9)	0.012
Current smoking				
No	Reference			
Yes	1.4 (1.0-1.9)	0.048	1.7 (1.2-2.6)	0.004
History of hypertension				
No	Reference			
Yes	1.2 (0.9-1.5)	0.134	0.9 (0.6-1.3)	0.975
Diabetes Mellitus				
No	Reference			
Yes	1.7 (1.3-2.3)	< 0.001	1.02 (0.6-1.6)	0.913
Dyslipidemia				
No	Reference			
Yes	1.1 (0.8-1.4)	0.345		
History of CVD				
No	Reference			
Yes	1.1 (0.9-1.1)	0.052	1.08 (0.7-1.6)	0. 684
Mother’s History of CVD				
No	Reference			
Yes	1.1 (0.9-1.1)	0.468		
Systolic Blood Pressure				
≤ 140 mmHg	Reference			
> 140 mmHg	1.1 (0.8-1.5)	0.247	0.9 (0.7-1.3)	0.821
Diastolic Blood Pressure				
≤ 90 mmHg	Reference			
> 90 mmHg	1.1 (0.8-1.5)	0.413		
Total Cholesterol				
≤ 200 mg/L	Reference			
> 200 mg/L	0.9 (0.7-1.2)	0.550		
Triglyceride				
≤ 150 mg/L	Reference			
>150 mg/L	1.1 (0.8-1.5)	0.319		
High-Density Lipoprotein				
≤ 40 mg/L	Reference			
> 40 mg/L	0.9 (0.7-1.2)	0.610		
Fasting Blood Sugar				
≤ 126 mg/L	Reference			
> 126 mg/L	2.2 (1.6-2.9)	< 0.001	2.1 (1.3-3.1)	0.001
Beta-Blocker				
No	Reference			
Yes	1.1 (0.8-1.4)	0.367		
ACE Inhibitor				
No	Reference			
Yes	1.2 (0.8-1.8)	0.189	0.9 (0.6-1.3)	0.597
Dipyridamole				
No	Reference			
Yes	1.2 (0.7-2.2)	0.342		
Anti-Lipid Drugs				
No	Reference			
Yes	1.2 (0.8-1.8)	0.300		
Antidiabetic Drugs				
No	Reference			
Yes	2.0 (1.4-2.7)	< 0.001	1.1 (0.6-1.9)	0.645
Antihypertensive Drugs				
No	Reference			
Yes	1.3 (1.1-1.8)	0.012	1.5 (1.07-2.1)	0.014

CVD, Cardiovascular disease; ACE inhibitor, Angiotensin-converting enzyme inhibitor

## Discussion

This study used the multiple Cox regression analysis to identify the factors contributing to the risk of recurrent CVD events in patients with established CVD. Our results showed that after controlling confounding variables, the most effective variables allied to CVD recurrence were respectively as follows: increased fasting blood sugar, smoking, taking antihypertensive drugs, male sex, taking antidiabetic drugs, father’s history of CVD, and history of diabetes. Fortunately, the most important variables influencing the recurrence of CVD were increased fasting blood sugar and smoking, both of which are modifiable variables. The results also revealed that the risk of CVD recurrence increased with age in both sex groups in the 1st 5 years of the follow-up, although the increase among the women was higher than that among the men (7.9 vs. 4.2). Further, diabetes and sex had a strong relationship with CVD recurrence, as is clearly shown in Figure 4. Diabetes had a very strong relationship with CVD recurrence in the 1st year. This relationship in the last year was detected more pronounced in the men than in the women. The possible reasons for the observed differences in the diabetic women versus the diabetic men at the end of the follow-up might have been good compliance with the physicians’ instructions and proper diets by the women in comparison with the men.

Although our study showed the impacts of the variables of diabetes and gender on the recurrence of CVD, the results would have been bolstered had there been a method to measure the effects of several variables on the recurrence of CVD simultaneously (such as the simultaneous effects of hypertension, smoking, and lipid disorders).

We highlighted the features that could be useful in both clinical practice and public health. First, increasing age had a significant relationship with CVD recurrence; this finding was also reported by Marmor et al.^11^ It is worthy of note, however, that patients who develop CVD at a young age often have different risk profiles as compared to older patients.^12^ Generally in Iran, life expectancy is not very high. As can be seen in Figure 3, in the last years of the follow-up, the risk of recurrence in the age group 60–90 years old was lower in the men than in the women, which could be the leading cause of death in older men. Of course the small sample size in this age group might be another reason. Second, the risk of recurrence was higher in the men than in the women (45%). Our result was similar to that of a study by Giorda et al.^13^ This result might have been obtained due to greater predisposition to necrotic events in men than in post-menopausal women, who are more prone to myocardial infarction, angina and, probably, heart failure.^14^^, ^^5^ Interestingly, we observed that there was no significant relationship between a history of CVD and the recurrence of CVD. This finding is inconsistent with that reported by Mulders et al.^16^ On the other hand, it is believed that standard treatment should preventthese recurrent events.^17^^, ^^18^ 

 We indicated that there was no significant relationship between a history of diabetes and the recurrence of CVD, while Deedwania et al.^19^ found a significant relationship between a history of diabetes and CVD recurrence. The disagreement might be explained by the differences in the study populations, study designs, follow-up durations, sample sizes, and statistical analyses. Indeed, Deedwania et al.^19^ investigated the relationship between diabetes and the recurrence of CVD by using the Cox regression method in addition to some other statistical methods such as the *t*, χ^2^, Kaplan–Meier, and Wilcoxon tests. The results of our study did not find a significant relationship between a history of diabetes and the recurrence of CVD, but a significant relationship was found between increased fasting blood sugar and the disease recurrence. Be that as it may, stating that these variables might play an important role in the recurrence of CVD should be carefully interpreted because we did not investigate the duration of suffering from diabetes as well as other medical disorders associated with this disease (e.g., nephropathy and retinopathy) due to the lack of relevant data, which might have confounded our results.

This study has several strengths and limitations. The most salient strength is that it is the 1st study to assess the factors associated with CVD recurrence in Iran. The long-term follow-up in the current study is another strong point. Furthermore, the data were extensive inasmuch as the study population was comprised of 483 cases. Also, the fact that there was a committee to confirm the deaths caused by CVD (not only the cause of death but also the cause of any event leading to hospitalization) can be deemed a strength. On the other hand, myocardial infarction is of various types, but a misclassification appeared in this study due to the lack of adequate information about the different types of myocardial infarction and the statistical results were diluted. Moreover, the outcome of myocardial infarction and angina as well as the outcome of strokes was integrated in 1 outcome (i.e., CVD), precluding a separate evaluation of the relationship between different exposures and outcomes. Indeed, it would have been preferable had the 2 groups not been integrated in the same outcome. Another noteworthy weakness is that the definition of smoking, according to the definition provided when completing the questionnaire, supposed that current smokers and former smokers (ex-smokers) had the same risk of death. Consequently, a misclassification occurred, which diluted the obtained result. This caused the observed risk to be lower than the real one. Also, the variable of low-density lipoprotein was excluded because the data related to it had not been collected. This variable can affect the recurrence of CVD.

## Conclusion

The results of the present study demonstrated that the established risk factors for the development of CVD (i.e., increased fasting blood sugar, smoking, taking antihypertensive drugs, masculinity, taking antidiabetic drugs, father’s history of CVD, and history of diabetes) played an important role in the risk of CVD recurrence. These results can help clinicians and patients as well as public health decision-makers to raise awareness about CVD recurrence. We showed that male sex was a strong risk factor for CVD recurrence compared to female sex, and this might be clinically important. Vis-à-vis smoking, the risk of CVD recurrence was higher in the smokers than in the nonsmokers. This finding can be useful to both clinical and public health applications regarding the prevention of CVD recurrence. 

## Notes:

***This paper should be cited as:*** Taravatmanesh S, Khalili D, Khodakarim S, Asgari S, Hadaegh F, Azizi F, Sabour S. Determining the Factors Associated with Cardiovascular Disease Recurrence: Tehran Lipid and Glucose Study. J Teh Univ Heart Ctr 2017;12(3):107-113.
